# Managing Acute Urinary Dysfunction for Neurologic Injury Patients

**Published:** 2020-05-19

**Authors:** Brandon Lucke-Wold

**Affiliations:** Department of Neurosurgery, University of Florida, Gainesville, FL, United States

## Background

Damage to the nervous system can have direct and indirect impact on the lower urinary tract. Broadly speaking, damage can be grouped into three categories: problems with bladder storage, stress incontinence, and problems with bladder emptying [1]. Many patients require bladder catheterization and are at increased risk for development of urinary tract infections [2]. Historically, urinary management has primarily involved urinary catheter bundles, but these may not be effective for the neurologic patient [3]. In this commentary, we highlight the anatomic basis behind neurologic injury in section 1 and then in section 2 offer a step-by-step clinician guide for managing neurologic dysfunction in the neurologic injury patient. Finally, in section 3 we provide key practice points per injury type.

## Section 1: Anatomic Basis of Injury

Bladder storage is mediated by signals from the pontine storage center to the pudendal nerve. In conjunction with sympathetic input, striated urethral sphincter activity increases the ability of the bladder to fill and maintain urine [4]. The process of micturition involves a coordinated response from smooth and striated muscles throughout the bladder and bladder neck. The process is mediated from bladder pressure input signals that transmit to the periaqueductal grey [5]. These fibers then trigger the pontine micturition center. In conjunction with the parasympathetic nerves, the stimulated pelvic nerve initiates micturition [6]. The pontine storage and micturition centers can be modulated from three distinct pathways. The first involves insula input to the periaqueductal grey. The second involves the prefrontal cortex to the anterior cingulated to hypothalamus and then to periaqueductal grey. Final pathway involves prefrontal cortex to basal ganglia and then to periaqueductal grey. Broadly speaking, brain injury can damage the frontal lobe connection pathways, pontine micturition centers, or periaqueductal grey [7].

## Section 2: Clinical Management Strategy by Injury Type

The below algorithm serves as a management strategy for tackling urinary dysfunction in the neurologic injury patients:

Step 1: Obtain clinically relevant imaging to determine primary site of injury (frontal lobe, pontine, periaqueductal grey).

Step 2A: If frontal lobe damage patient likely to have urinary frequency and/or incontinence (problems with bladder storage). Initiate urodynamic studies, start anticholinergics, and engage psychology colleagues to help with behavioral therapy.

Step 2B: If pontine micturition center damaged patient likely to have inability to void and/or increased post-void residuals (problems with bladder emptying). Initiate foley, start tamsulosin, and begin bladder training. If foley cannot be weaned, initiate urology consult for evaluation of detrusor stimulator.

Step 2C: If periaqueductal grey damaged patient likely to have stress incontinence [8]. Initiate psychology consult. Encourage drinking fluids at a consistent time followed by scheduled urination. Initiate exercise regimen and encourage weight loss.

Step 3: Schedule outpatient follow up with multidisciplinary team including: neurologist, primary care physician, physical and occupational therapists, and clinical psychologists.

## Section: Practice Points Based on Injury Type

Traumatic brain injury: acutely causes increased renal perfusion leading to increased frequency of voiding. This is counter acted over time by increased collagen deposition in the bladder [9]. In the acute recovery period, these patients often will require in and out catheterization until bladder collagen develops.

Hemorrhagic stroke: underlying hypertension causes kidney damage [10]. These patients are more susceptible to high residual volumes and delayed emptying times. Foley catheters are often required for these patients.

Ischemic stroke: ramps up the sympathetic system causing acute urinary frequency followed by urinary retention [11]. This is due to sphincter dysynergy. These patients would benefit from bladder training exercises.

Subarachnoid hemorrhage: early urinary retention is frequently seen [12]. Reducing catecholamines may be beneficial as a treatment modality.

Subdural hematoma: often causes acute urinary retention followed by overflow incontinence [13]. Treating underlying subdural hematoma often improves urinary function.

Epilepsy: urinary urgency is seen prior to seizure with urinary incontinence often during the seizure [14]. Todd’s paresis can subsequently be associated with urinary retention. Preventing further seizures normally helps restore urinary equilibrium.

## Summary

Urinary dysfunction following neurologic injury warrants careful investigation. Going forward, it will be imperative to switch from the urinary bundle model to the more appropriate disease specific modality treatment. This type of approach utilizes not only anatomical understanding but also patient specific factors. The ideal environment includes a multidisciplinary team involving clinicians, therapists, and nursing staff. As the patient is discharged into the community, it is important that family members be instructed on symptoms to watch out for in regards to urinary dysfunction, what resources are available, and how they can help their loved one. Public health initiatives will be required to improve knowledge within the community and increase overall acceptance of engineering changes that can be implemented to assist these patients. Some of these changes may include updated restrooms that are easier to use for patients with urinary frequency, visual reminders for the need of urination throughout the day, and training regarding self-catheterization.

In conclusion, muscle weakness occurs in numerous ME/ CFS patients limiting their capacities at work. The causes of muscle weakness in ME/CFS are not fully understood and perhaps combine peripheral and central fatigue. Because measurement of maximal exercise capacities on a cycloergometer or a treadmill is often difficult in severely fatigued patients, the simple determination of maximal handgrip strength can be useful to evaluate the limitation of physical performance.
